# The anticoagulation length of therapy and risk of new adverse events in venous thromboembolism (ALTERNATIVE) study: Design and survey results

**DOI:** 10.1371/journal.pone.0277961

**Published:** 2022-12-08

**Authors:** Cecilia Portugal, Margaret C. Fang, Alan S. Go, Hui Zhou, John Chang, Priya Prasad, Dongjie Fan, Elisha A. Garcia, Sue Hee Sung, Kristi Reynolds

**Affiliations:** 1 Department of Research and Evaluation, Kaiser Permanente Southern California, Pasadena, CA, United States of America; 2 Division of Hospital Medicine, University of California, San Francisco, San Francisco, CA, United States of America; 3 Division of Research, Kaiser Permanente Northern California, Oakland, CA, United States of America; 4 Department of Health Systems Science, Kaiser Permanente Bernard J. Tyson School of Medicine, Pasadena, CA, United States of America; 5 Department of Medicine and Department of Epidemiology, and Biostatistics, University of California, San Francisco, San Francisco, CA, United States of America; 6 Departments of Medicine, Stanford University, Palo Alto, CA, United States of America; Ataturk University Faculty of Medicine, TURKEY

## Abstract

The Anticoagulation Length of Therapy and Risk of New Adverse Events In Venous Thromboembolism (ALTERNATIVE) study was designed to compare the benefits and harms of different treatment options for extended treatment of venous thromboembolism (VTE). In this paper, we describe the study cohort, survey data collection, and preliminary results. We identified 39,605 adult patients (age ≥ 18 years) from two large integrated health care delivery systems who were diagnosed with incident VTE and received initial anticoagulation therapy of 3 months or longer. A subset of the cohort (12,737) was invited to participate in a survey. Surveys were completed in English, Spanish or Mandarin via a mailed questionnaire, an online secure web link, or telephone. The survey domains included demographics, personal medical history, anticoagulant treatment history, anticoagulant treatment satisfaction, health-related quality of life and health literacy. A total of 5,017 patients participated in the survey for an overall response rate of 39.4%. The mean (SD) age of the survey respondents was 63.0 (14.5) years and self-reported race was 76.0% White/European, 11.1% Black/African American, and 3.8% Asian/Pacific Islander and 14.0% reported Hispanic ethnicity. Sixty percent of respondents completed the web survey, while 29.0% completed the mail-in paper survey, and 11.0% completed the survey via telephone. The ALTERNATIVE Study will address knowledge gaps by comparing several treatment alternatives for the extended management of VTE so that this information could be used by patients and clinicians to make more informed, patient-centered treatment choices.

## Introduction

Venous thromboembolism (VTE), primarily pulmonary embolism (PE) and deep venous thrombosis (DVT), affects up to 1 million Americans annually, resulting in morbidity (e.g., post-thrombotic syndrome) and mortality that affects all races, ethnicities, age groups and genders [1, 2]. Anticoagulants, which reduce the ability of the blood to clot, are the medications most commonly prescribed to treat VTE [3]. Several anticoagulant options are available for acute and extended treatment of VTE [4–7]. These include oral vitamin K antagonists (primarily warfarin) and direct oral anticoagulants (DOACs) including dabigatran, rivaroxaban, apixaban, and edoxaban [8–10]. DOACs offer advantages over warfarin such as fewer drug-drug interactions, fewer dietary restrictions, less routine lab monitoring and standardized drug dosing [11]. However, DOACs are more costly and like all anticoagulants, can increase the risk of bleeding [12]. Although the American Society of Hematology 2020 guidelines for management of VTE recommend primary treatment of a 3–6 month regimen of anticoagulant treatment after diagnosis of VTE, patients with a higher risk of VTE recurrence are often prescribed extended or lifelong anticoagulation therapy [13–15]. However, there is limited real-world evidence regarding optimal duration of anticoagulant therapy for VTE in terms of risks and benefits as extending the duration of anticoagulation will significantly increase bleeding risk which can be detrimental especially for patients of advanced age [16–19].

The **A**nticoagulation **L**ength of **T**h**E**rapy and **R**isk of **N**ew **A**dverse even**T**s **I**n **V**enous thrombo**E**mbolism (ALTERNATIVE) study was funded by the Patient-Centered Outcomes Research Institute (PCORI) to compare the benefits and harms of different treatment options for extended treatment of VTE. The ALTERNATIVE study was a retrospective new-user cohort of patients with VTE that combined data from a cross-sectional survey and the electronic health record (EHR). We conducted a survey to assess patients’ treatment satisfaction with anticoagulants, health literacy, and quality of life. In this manuscript, we provide an overview of the ALTERNATIVE cohort and describe the survey design, methods, and preliminary results.

## Methods

### Study setting and surveyed population

The study was approved by the Institutional Review Board (IRB) of Kaiser Permanente Northern California (KPNC). Kaiser Permanente Southern California ceded IRB to KPNC. The source populations for the ALTERNATIVE study were members of Kaiser Permanente Northern California (KPNC) and Kaiser Permanente Southern California (KPSC), two large, integrated health care delivery systems that provide comprehensive care for > 9 million diverse members across California. Members are highly representative of the statewide population except for slightly lower representation at the extremes of income and age [20, 21]. All aspects of care are captured through the KP HealthConnect™ electronic medical record (Epic Systems, Verona, WI). The primary data source for the overall cohort assembly and characterization was from the Virtual Data Warehouse (VDW) [22]. Data elements from the VDW included demographics (age, gender, race and ethnicity), medical history (prior VTE, thrombophilia, hypertension, dyslipidemia, diabetes, cancer, chronic kidney disease, chronic liver disease, intracranial hemorrhage, peptic ulcer disease), medications (oral anticoagulants, angiotensin-converting enzyme inhibitors, angiotensin II receptor blockers, beta-blockers, calcium channel blockers, diuretics, aldosterone receptor antagonists, non-aspirin antiplatelet agents, and lipid-lowering agents), lab test results (hemoglobin, hematocrit, platelet, serum creatinine), clinical encounters (hospital, emergency department, outpatient), diagnostic tests for VTE and CT tests (chest, lung, inferior vena cava), death date (health plan records).

The population of patients eligible for the survey was first identified using administrative and clinical data. We first identified adult (age ≥ 18 years) patients diagnosed with incident VTE between January 1, 2015, to December 31, 2018, and who had continuous outpatient anticoagulant therapy for ≥ 3 months after the incident VTE diagnosis. An incident VTE diagnosis was defined as having ≥1 outpatient encounter, hospitalization, or emergency department visit associated with either a primary or secondary discharge diagnosis code of VTE according to the International Classification of Diseases, Ninth Revision, (ICD-9) or Tenth Revision (ICD-10) and without prior use of anticoagulants in the previous 4 years. Patients were excluded if they had missing information on age or gender, had < 12 months of continuous health plan membership or pharmacy benefits prior to the VTE index date, had a prior diagnosis of VTE or anticoagulant dispensed within 4 years before the index VTE date. To be eligible for the survey, patients were alive at the time of the survey, active health plan members, with a valid mailing address, email, or telephone number, and whose primary language was English, Spanish, or Mandarin Chinese.

### Survey development and questions

The survey was developed by the study research investigators and a Steering Advisory Committee (SAC) that was comprised of stakeholders who represented various perspectives of VTE treatment. Stakeholder representatives included the National Blood Clot Alliance (NBCA, a thrombosis-oriented patient-advocacy organization), clinicians with expertise in anticoagulation, representatives of each participating health system, and patients/caregivers with personal experience with VTE. The primary purpose of the survey was to obtain patient-level information not routinely available from the EHR including certain sociodemographic characteristics, awareness of medical history and family history of thrombosis, health literacy and patient-reported outcomes, specifically, health-related quality of life (QOL) and anticoagulation treatment satisfaction (Table 1). We pilot tested the survey in 18 volunteer patients with a history of VTE who were recruited through the NBCA. The findings from the pilot test showed that the survey took approximately 10-minutes to complete and was rated as easy to complete. We incorporated suggestions to improve the clarity of the survey when feasible. The final ALTERNATIVE survey was 10 pages in length and contained 72 close-ended questions.

**Table 1:** Survey domains and measurements obtained in the ALTERNATIVE survey cohort.

Domain	Measurement
**Demographics**	Race
	Hispanic ethnicity
	Preferred language
	Education level
	Household income
	Marital status
**Medical History**	Prior VTE (deep vein thrombosis and/or pulmonary embolism)
	Atrial fibrillation
	Heart valve replacement
	Hypercoagulable condition
	Family history of thrombosis
**Anticoagulant Treatment History**	Prescription for anticoagulants (warfarin, DOACs)
	Therapeutic change of oral anticoagulants and reasons for change
	Bleeding complications from anticoagulants
	Early discontinuation of anticoagulants and reasons why
	Exposure to non-prescription medications that increase bleeding risk (e.g., aspirin, NSAIDS)
**Health-related Quality of Life**	SF-36 instrument [23]
	• Self-rated health
	• Physical functioning scale
	• Role limitation physical scale
	• Role limitation emotional scale
	• Energy fatigue scale
	• Emotional well-being scale
	• Social functioning scale
	• Pain scale
	• Physical component summary
	• Mental
**Anticoagulation Treatment Satisfaction**	ACTS instrument [24]
	• Benefit scale
	• Burden scale
**Health Proficiency**	Health literacy [25, 26]

For those who were actively taking oral anticoagulants (DOACs or warfarin) for incident VTE occurring between January 1, 2015 and June 30, 2018, treatment satisfaction was measured using the Anti-Clot Treatment Scale© (ACTS) [24], which is the most rigorously developed and psychometrically-tested patient-reported scale for anticoagulation treatment satisfaction. The ACTS scale is a 17-item instrument, comprising 13 burden items (assessing challenges with anticoagulation) and 4 benefit items (assessing confidence and reassurance in anticoagulation). Each item is scored on a 5-response Likert scale. The ACTS scale has two sub-scales: ACTS Burdens and ACTS Benefits. ACTS Burdens is reverse coded on a 5-point Likert scale and is the sum of 12 items (range 12–60). ACTS Benefits is coded from 1 to 5 and is the sum of 3 items (range 3–15). Higher scores denote greater satisfaction with treatment [24, 27, 28].

Quality of life (QOL) was assessed using the validated 36-Item Short Form survey [SF-36] from the Medical Outcomes Study [23]. The SF-36 is scored to create eight scales: physical functioning (PF), role physical (RP), bodily pain (BP), general health (GH), vitality (VT), social functioning (SF), role emotional (RE), and mental health (MH) [29, 30]. Physical component summary (PCS) and mental health component summary (MCS) scores were derived from the 8 scales according to the user’s manual [23]. Specifically, three steps were performed to obtain the PCS and MCS components including 1) standardize the 8 SF-36 scales using a z-score transformation from the general U.S population; b) computing aggregated PCS and MCS scores by multiplying each SF-36 scale z-score by the respective factor coefficient using formulas provided; 3). transforming the aggregated score to the norm-based scoring by multiplying the score by 10 and adding the resulting product to 50. SF-36 domain scores range from 0–100, with higher scores indicating better QOL.

Health literacy was assessed using a validated health literacy tool (“3 Health Literacy Questions”) to assess patient understanding of medical information [25, 26]. The questions were scored via a five-point Likert scale (0–4 scale for each question (0 = most literate, 4 = least literate); the higher score values represent lower literacy [25]. Scoring, including imputations for missing data if necessary, was performed according to each instrument developer’s guidelines.

### Patient recruitment

The survey was administered by ANA Research (Anderson, Niebuhr & Associates, Inc.), a market research company. The survey was conducted in two waves: wave 1 in 2018 (included VTE events from January 1, 2015, through April 2017) and wave 2 in 2019 (included VTE events from May 2017 through December 31, 2018). All eligible patients were mailed a survey packet (invitation letter, survey, and postage-paid opt-out postcard) in English and in their preferred language of Mandarin or Spanish. The invitation letter was printed on Kaiser Permanente letterhead, introduced ANA as the research partner and described the study. Patients were given the opportunity to complete the survey on a paper form, by telephone or online via a secure web link. Additionally, an email invitation, in both English and their preferred language, was sent to those with a valid email address. A second mailing (in wave 1 only) was sent to non-respondents approximately 14 days after the initial mailing and included another cover letter and survey. Reminder emails were sent to non-respondents one week and three weeks after the initial email. In wave 1, patients who did not respond to either mail or email received a follow-up telephone call to complete the survey by phone. Up to nine call attempts were made with a maximum of three messages over three continuous weeks. For wave 2, we surveyed patients with a VTE diagnosis between May 2017 and December 31, 2018, via the web survey and to ensure adequate representation of DOAC users, we mailed survey packets to those who did not respond to the web survey or did not have a valid email address. No telephone interviews were conducted during wave 2. Respondents were mailed a $25 Visa gift card for their time spent completing the survey.

### Statistical analysis

Means and standard deviations (SD) for continuous variables or percentages for categorical variables were used to describe patient characteristics. We compared demographic and VTE clinical characteristics obtained from the EHR between respondents and non-respondents, using Cohen’s *D* value from the standardized difference in means or proportions between the two groups. We considered a value of d >0.2 as a meaningful difference [31]. We examined the demographic characteristics of respondents by each survey mode (mail, email, telephone). We calculated mean (SD) summary scores for the patient reported outcomes. All analysis were conducted using SAS Enterprise Guide 9.3 (SAS Institute, Cary, NC).

## Results

### ALTERNATIVE survey cohort

**Fig 1:**
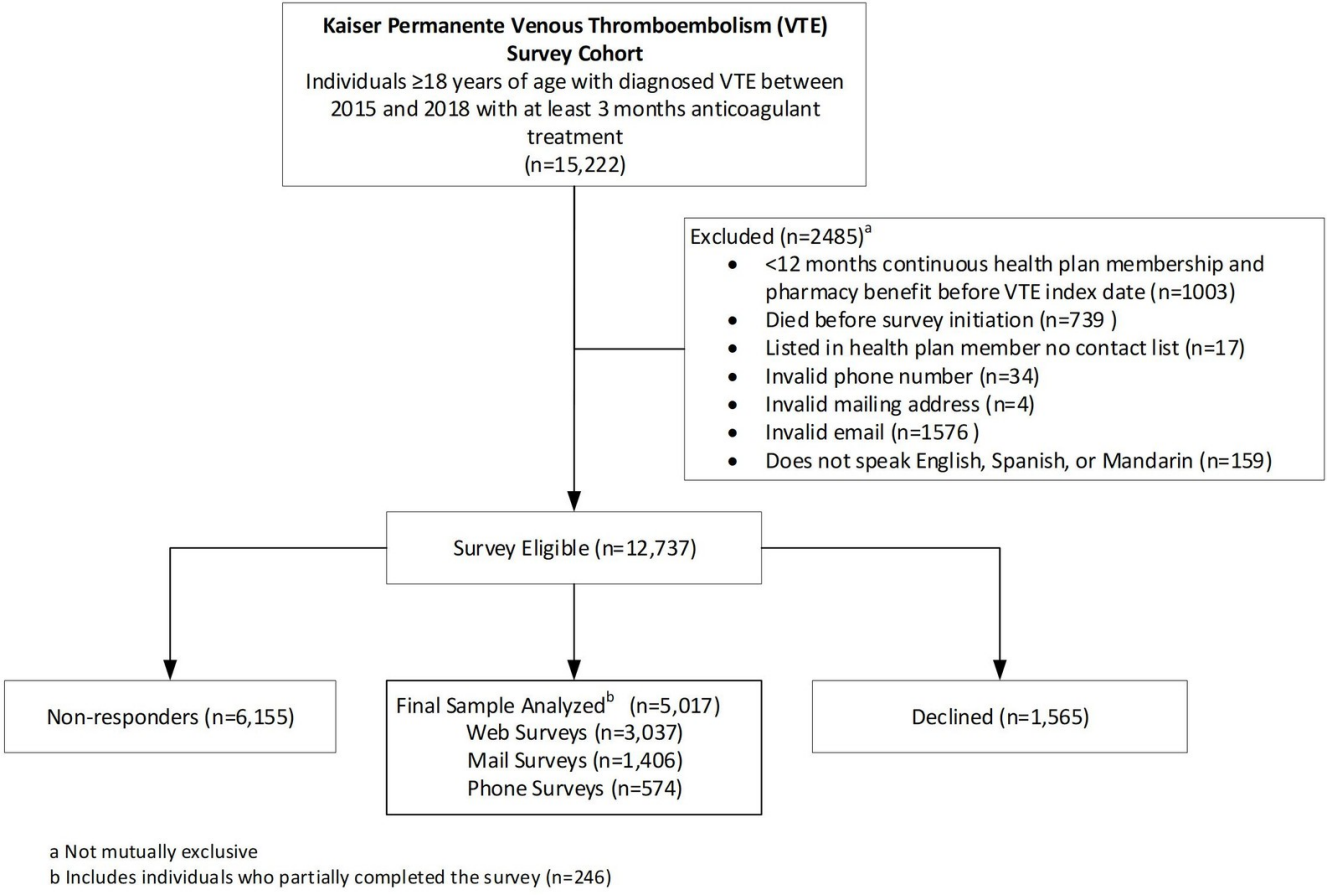
Survey cohort flow diagram. a) Not mutually exclusive. b) Includes individuals who partially completed the survey (n = 246).

A total of 12,737 were eligible and invited to complete the survey. Among them, 1,565 declined to participate and 6,155 did not respond, leaving 5,017 people who completed some or all of the survey (Fig 1). Two-hundred and forty-six patients only completed part of the survey. The overall survey response rate was 39.4%. There were no statistically significant differences in the characteristics of respondents and non-respondents (Table 2). The mean (SD) age of respondents was 62.7 (15.9) years, 48.9% were female, 69.7% were White adults, 14.0% Black/African American adults, 5.2% Asian/Pacific Islander adults and 17.5% Hispanic ethnicity. The majority of VTE events were identified in the emergency department (47.1%) followed by inpatient (33.9%) and outpatient setting (19.1%), with 45.7% of incident VTE events being PE and 43.4% being lower extremity DVT.

**Table 2:** Baseline characteristics of respondents and non-respondents in the ALTERNATIVE survey cohort.

Variable	Overall	Respondents*	Non-Respondents	D-Value±
	(N = 12,737)	(N = 5,017)	(N = 7,720)	
**Age, years, mean (SD)**	62.7 (15.9)	63.0 (14.5)	62.5 (16.6)	0.03
**Age group, years, n (%)**				0.11
≤54	3668 (28.8)	1292 (25.8)	2376 (30.8)	
55–64	2759 (21.7)	1178 (23.5)	1581 (20.5)	
65–74	3139 (24.6)	1442 (28.7)	1697 (22.0)	
75–84	2283 (17.9)	860 (17.1)	1423 (18.4)	
≥ 85	888 (7.0)	245 (4.9)	643 (8.3)	
**Female, n (%)**	6230 (48.9)	2478 (49.4)	3752 (48.6)	0.01
**Race, n (%)**				0.11
White/European	8881 (69.7)	3815 (76.0)	5066 (65.6)	
Black/African American	1784 (14.0)	553 (11.0)	1231 (15.9)	
Asian/Pacific Islander	657 (5.2)	190 (3.8)	467 (6.0)	
Other	63 (0.5)	27 (0.5)	36 (0.5)	
Unknown	1352 (10.6)	432 (8.6)	920 (11.9)	
**Hispanic ethnicity, n (%)**				0.07
Hispanic	2227 (17.5)	702 (14.0)	1525 (19.8)	
Non-Hispanic/Unknown	10,510 (82.5)	4315 (86.0)	6195 (80.2)	
**VTE encounter type, n (%)**				0.04
Outpatient	2427 (19.1)	1006 (20.1)	1421 (18.4)	
Emergency Department	5998 (47.1)	2415 (48.1)	3583 (46.4)	
Inpatient	4312 (33.9)	1596 (31.8)	2716 (35.2)	
**VTE type, n (%)**				0.05
Pulmonary Embolism	5818 (45.7)	2387 (47.6)	3431 (44.4)	
Lower Extremity Deep Vein Thrombosis	5530 (43.4)	2168 (43.2)	3362 (43.5)	
Upper Extremity Deep Vein Thrombosis	757 (5.9)	234 (4.7)	523 (6.8)	
Mesenteric Venous Thrombosis	305 (2.4)	111 (2.2)	194 (2.5)	
Unknown VTE	327 (2.6)	117 (2.3)	210 (2.7)	
**Site, n (%)**				0.03
KPNC	6010 (47.2)	2454 (48.9)	3556 (46.1)	
KPSC	6727 (52.8)	2563 (51.1)	4164 (53.9)	
**Index year, n (%)**				0.12
2015	3354 (26.3)	1453 (29.0)	1901 (24.6)	
2016	3736 (29.3)	1702 (33.9)	2034 (26.4)	
2017	3930 (30.8)	1333 (26.6)	2597 (33.6)	
2018	1717 (13.5)	529 (10.5)	1188 (15.4)	

*Includes 246 individuals who partially completed surveys.

**±**Values >0.2 are considered meaningfully different

Most surveys were completed online (n = 3037; 60%), followed by mail/paper survey (n = 1406; 29%) and telephone (n = 574; 11%) (Table 3). This finding was consistent across age, sex, and race-ethnicity groups except for the 75–84 and ≥85-year age groups where a majority completed their survey by mail, followed by web and telephone.

**Table 3:** Survey completion by survey mode and self-reported race/ethnicity.

Variable	Total (N = 5017)	Web (N = 3037)	Mail (N = 1406)	Phone (N = 574)	p-value
**Age, years, n (%)±±**					<0.0001
≤54	1112 (22.2)	727 (23.9)	178 (12.7)	207 (36.1)	
55–64	1062 (21.2)	695 (22.9)	243 (17.3)	124 (21.6)	
65–74	1460 (29.1)	922 (30.4)	402 (28.6)	136 (23.7)	
75–84	1023 (20.4)	537 (17.7)	400 (28.5)	86 (15.0)	
> = 85	360 (7.2)	156 (5.1)	183 (13.0)	21 (3.7)	
**Gender, n (%)±±**					0.3382
Female	2478 (49.4)	1479 (48.7)	701 (49.9)	298 (51.9)	
Male	2539 (50.6)	1558 (51.3)	705 (50.1)	276 (48.1)	
**Race Ethnicity, n (%)**					<0.0001
White/European	3260 (65.0)	2069 (68.1)	934 (66.4)	257 (44.8)	
Black/African American	468 (9.3)	260 (8.6)	125 (8.9)	83 (14.5)	
Asian or Pacific Islander	137 (2.7)	80 (2.6)	41 (2.9)	16 (2.8)	
Hispanic	642 (12.8)	299 (9.8)	187 (13.3)	156 (27.2)	
Other	254 (5.1)	146 (4.8)	67 (4.8)	41 (7.1)	
Unknown	256 (5.1)	183 (6.0)	52 (3.7)	21 (3.7)	

**±**Race Ethnicity from survey self-report

**±±**Obtained from VDW EHR data

#### Anticoagulant history

Among all survey respondents, 16.9% (n = 850) reported a family history of VTE and 29.6% (n = 1487) reported anticoagulant use within the past 4 weeks. Among those who reported a history of anticoagulant use (N = 4,773), 16.5% (n = 787) reported changing anticoagulants; the most common reasons were convenience (31.8%, n = 251) and side-effects (23.5%, n = 185). Over 10% of patients (n = 497) with a history of anticoagulant use reported a history of bleeding and sought medical attention but did not require hospitalization while on oral blood thinners.

There were 2,224 patients defined as active anticoagulation users who reported recent anticoagulant use within the 4 weeks prior to the survey. Among them, 11.8% (N = 262) reported a history of bleeding that required medical assistance and 22.0% (n = 489) reported a use of antiplatelets. The patient reported outcomes among those who were on active anticoagulation are presented in Table 4. Mean (SD) ACTS Burden and Benefits scores were 52 (8.1) and 10 (3.0), respectively, compared to the reference range for each subscale (12–60 and 3–15, respectively) indicating satisfaction with anticoagulation treatment. The SF-36 mental health component mean score was 75 and SD of 19, while the physical component score had a mean of 42 with SD of 12. Over 17% of the survey respondents were categorized as having inadequate health literacy.

**Table 4:** Summary scores of the patient-reported outcomes and health literacy.

Scale and Domain	Mean (SD)
**ACTS (N = 2,227)**	
Burden	52 (8.1)
Global burden	4 (1.0)
Benefit	10 (3.0)
Global benefit	3 (1.2)
**SF-36 (N =** 4,776**)**	
Physical functioning	66 (31.6)
Role Physical	59 (43.3)
Bodily Pain	65 (27.0)
General Health	59 (22.3)
Vitality	52 (23.2)
Social Function	75 (27.9)
Role Emotional	73 (38.7)
Mental Health	75 (18.8)
Physical component	42 (12.4)
Mental health component	50 (10.8)
**Health Literacy**	**N (%)**
Adequate	3,946 (82.8)
Inadequate	820 (17.2)

## Discussion

In the ALTERNATIVE study survey of 12,737 patients with VTE, a higher proportion of patients completed the survey by the web, followed by mail and telephone. This was consistent across age, sex, and race-ethnic groups except for patients ages 75 and older who preferred mail followed by web and telephone. Survey respondents were similar to non-respondents in regard to demographics and VTE type.

One of the successes of this large prospective data collection study was offering the mixed mode survey options (mail, web-based and telephone) thus enabling greater survey participation according to convenience, preference, and needs. In our study, the web-based platform was the most cost-effective mode compared to the expense of mailing out survey packets and the more costly telephone survey administration mode. Furthermore, the web-based data had fewer missing data due to built-in skip patterns and the option for respondents to be able to stop a survey at any time and restart it later where they had previously left off, which may have led to less survey fatigue. The web-based survey data collection was less labor intensive, avoided manual data entry, and rendered the highest response rate, which is contrary to the findings of other survey data collections studies [32–36]. In the overall cohort, the highest mode of survey participation was web-based and half were age 65 years and older. However, when looking at age by survey mode, a majority of the mail survey participants were age 65 years or older which is a trend found in other studies regarding older participants preferring mail surveys [36, 37]. Offering the mixed mode survey options in addition to the survey in three languages (English, Spanish and Mandarin) helped to meet the needs of our diverse patient population. Overall, the demographic features and type of VTE were similar between respondents vs. non-respondents, supporting that respondents were likely reflective of the source incident VTE population.

Several limitations of our study should be taken into consideration. Our cohort consisted of insured patients in California; therefore, the study results may not be fully generalizable to uninsured populations, health systems, or geographic areas. Further, it is possible that the experiences of the patients who responded to the survey do not fully represent the perspective of the overall VTE population. There is a possibility that some of the web-based survey invitations landed in spam folders where they went unnoticed and thus negatively impacted the web-based response rates. Lastly, although the letter invitations were sent out on KP letterhead with KP contact information, some members reached out to study staff and were wondering why an outside vendor (ANA) was requesting survey data; this could have caused a hesitation in survey participation for some individuals.

Offering a $25 gift card for survey participation in our study was a plus as other studies have shown that offering incentives is effective in increasing survey participation rates [38]. The strengths of this study include the racial/ethnic diversity, the survey being offered in different languages and different modes of administration. For Spanish or Mandarin-speaking health plan members, we utilized language preferences that were flagged in the electronic health record to help guide survey language options; these individuals were then able to choose their preferred survey language (English, Spanish, Mandarin). This language option been shown to be an effective method to increase survey participation among Hispanics [39].

## Conclusion

The goal of the ALTERNATIVE study is to compare the effectiveness and safety of anticoagulation therapy for VTE. To enrich the ALTERNATIVE study cohort data, we conducted a survey to assess patients’ treatment satisfaction with anticoagulants, health literacy, and quality of life. The findings from the survey add to previous research on the use of mixed mode survey administration in cohorts that are diverse in age, gender, preferred language, and race/ethnicity. Methods of survey administration (mail, telephone, and email) differ in missing data, cost, burden on respondent, access, or comfort with digital platforms for online surveys, and will continue to be important factors in survey research. It is suggested that future research give special attention to strategies that improve overall survey participation rates, such as, offering surveys in spoken/preferred language, providing incentives for participation, and offering surveys through different modalities (phone, paper/mail and web-based).
